# Body Mass Index and Sporadic Medullary Thyroid Cancer: Insights from a Large Series

**DOI:** 10.3390/cancers17060950

**Published:** 2025-03-11

**Authors:** Alessandro Prete, Carla Gambale, Valeria Bottici, Virginia Cappagli, Giacomo Aringhieri, Marco Puccini, Stefano Landi, Liborio Torregrossa, Ferruccio Santini, Antonio Matrone, Rossella Elisei

**Affiliations:** 1 Unit of Endocrinology, Department of Clinical and Experimental Medicine, University of Pisa, 56124 Pisa, Italy; alessandro.prete@phd.unipi.it (A.P.); carla.gambale@phd.unipi.it (C.G.); valeria.bottici@ao-pisa.toscana.it (V.B.); virginia.cappagli@med.unipi.it (V.C.); ferruccio.santini@unipi.it (F.S.); rossella.elisei@unipi.it (R.E.); 2 Academic Radiology, Department of Translational Research and New Technologies in Medicine and Surgery, University of Pisa, 56124 Pisa, Italy; giacomo.aringhieri@unipi.it; 3 Endocrine Surgery Unit, Department of Surgical, Medical and Molecular Pathology and Critical Care Medicine, University of Pisa, 56124 Pisa, Italy; marco.puccini@unipi.it; 4 Department of Biology, University of Pisa, 56124 Pisa, Italy; stefano.landi@unipi.it; 5 Pathology Unit 3, Department of Surgical, Medical and Molecular Pathology, University of Pisa, 56124 Pisa, Italy; liborio.torregrossa@unipi.it

**Keywords:** excess adipose tissue, medullary thyroid cancer, BMI, RET gene, RAS gene, calcitonin

## Abstract

**Background**: Excess adipose tissue has been associated with the increased prevalence and aggressiveness of many human cancers. While its role in differentiated thyroid cancer is well established, in medullary thyroid cancer (MTC), data are conflicting. We aimed to evaluate the impact of excess adipose tissue on MTC prevalence, aggressiveness at diagnosis, and outcome in a large series of patients. **Methods**: We evaluated 529 patients with sporadic MTC from a prospectively maintained database. Weight and height were measured in all patients at the time of surgery, and body mass index (BMI) was calculated. Therefore, patients were classified according to BMI categories suggested by the WHO for Caucasian patients. Data about somatic mutations were available in 254/529 patients (48.0%). **Results**: The prevalence of subjects with obesity was higher than that in the Italian general population (20.2 vs. 12.0%). Patients with obesity presented smaller tumors, lower T and N stage, and lower *AJCC 8th edition* stage, as well as lower preoperative calcitonin values compared to under/normal and overweight ones. The prevalence of somatic *RET* and *RAS* mutations did not differ significantly across the WHO BMI categories. At the end of follow-up, structural disease was less common in patients with obesity (15.4%) compared to under/normal (25.6%, *p* < 0.05) and overweight (24.1%, *p* = 0.079). Further surgeries for local recurrence were less common in patients with obesity (3.8%) compared to overweight ones (11.0%, *p* < 0.05). Survival was superimposable in the different categories of BMI. **Conclusions**: Among patients with sporadic MTC, there is a higher prevalence of patients with obesity compared to the general population. A higher BMI was associated with less aggressive clinical presentation and better clinical outcomes.

## Simple Summary

Excess adipose tissue has been linked to increased prevalence and aggressiveness in many cancers, but its role in medullary thyroid cancer (MTC) remains unclear. This study analyzed anthropometric and clinical data of a large series of patients with sporadic MTC to assess the impact of excess adipose tissue on MTC prevalence, aggressiveness, and outcome. Patients with obesity presented a less aggressive MTC compared to patients with normal weight or overweight, smaller tumors, lower T and N stages, and lower preoperative calcitonin levels. Somatic mutations occurring in *RET* and *RAS* genes did not differ across BMI categories. At the end of follow-up, patients with obesity showed less structural disease, resulting in a lower need for further surgical treatments. These findings suggest that patients with obesity experience less aggressive MTC, potentially due to increased medical examinations in this population.

## 1. Introduction

Excess adipose tissue is closely linked to several types of human cancers, such as colon, rectum, and breast cancers [1]. High body mass index (BMI), often used as a surrogate measure of adipose excess, has been associated with higher incidence of many cancers and poorer prognosis [2,3].

Thyroid cancer is the most common endocrine tumor, representing 3.4% of all cancers diagnosed annually [4]. Papillary (PTC), follicular (FTC), poorly differentiated (PDTC), and anaplastic (ATC) thyroid cancers arise from thyroid follicular cells, while medullary thyroid cancer (MTC) originates from parafollicular cells.

A significant association has been observed between higher BMI and higher thyroid cancer incidence, particularly PTC, while the potential influence of excess adiposity on MTC incidence is conflicting [5,6,7]. For instance, Engeland et al. showed a surprising inverse correlation between BMI and MTC risk, observing that MTC risk tended to decrease with increasing BMI [6]. This may suggest that adipose excess may influence follicular- and parafollicular-derived cancers differently based on their cellular origins.

Many studies have explored the link between BMI and PTC aggressiveness and prognosis. Some studies observed an increased aggressiveness in patients with obesity [8,9], while others did not [10,11]. Similarly, while some studies connect higher BMI with worse prognosis [5,12], others report no increased risk of persistence or recurrence in PTC patients with obesity [13,14]. In a large series of consecutive patients with differentiated thyroid cancer (DTC), we did not observe any association between BMI and aggressiveness of DTC, neither at the time of diagnosis nor during follow-up [15]. On the other hand, only a recent article addressed the potential correlation between BMI and MTC aggressiveness and prognosis. After the inclusion of both sporadic and hereditary MTC cases, they did not find any association between BMI and MTC aggressiveness [16]. Hence, to the best of our knowledge, no data are available about the potential correlation between BMI and sporadic MTC aggressiveness and prognosis.

The present study aimed to evaluate the prevalence of obesity, calculated by BMI, in a large cohort of patients with sporadic MTC. The correlation of obesity with MTC aggressiveness at diagnosis and its outcome was also evaluated.

## 2. Materials and Methods

### 2.1. Patients

From a prospectively maintained database, we evaluated 529 patients with sporadic MTC who were treated by surgery from January 2000 to December 2020 and followed at the Endocrine Unit of the University Hospital of Pisa up to the data lock of January 2024. The median age at diagnosis was 55 (interquartile range 45–66). The sporadic nature of the cases was assessed by a negative *RET* germline screening that is routinely performed in all cases of MTC in our hospital, according to the European Thyroid Association guidelines [17].

### 2.2. Anthropometric Data

Weight and height were measured in all patients at the time of surgery by the physicians, and BMI (kg/m^2^) was calculated as the body mass (Kg) divided by the square of the body height (m). Therefore, according to BMI categories suggested by the World Health Organization for Caucasian patients, our patients were divided into 4 groups: underweight (<18.5 kg/m^2^), normal weight (18.5–24.9 kg/m^2^), overweight (25.0–29.9 kg/m^2^), and obese (≥30.0 kg/m^2^).

### 2.3. Calcitonin (Ct) Assay

At each clinical evaluation, serum Ct was measured in our laboratory in all patients. Throughout the study period (2000–2024), we utilized three different Ct assays: from January 2000 to September 2013, an immunoradiometric assay (ELSA-hCT, CIS, Gif-Sur-Yvette, France—analytical sensitivity 2 pg/mL; normal reference ranges < 10 pg/mL for both sexes), from September 2013 to December 2020, a chemiluminescent immunometric assay (Immulite, Siemens Healthcare Diagnostic Products Ltd., Lianberis, Gwynedd, UK—analytical sensitivity 2 pg/mL; normal reference ranges < 18.2 pg/mL for males and <11.5 pg/mL for females), and from January 2021 to the data lock, an ultrasensitive chemiluminescent immunometric assay (Calcitonin II-Gen, DiaSorin LIAISON—analytical sensitivity 1 pg/mL and normal reference ranges < 18.2 pg/mL for males and <11.5 pg/mL for females).

### 2.4. Histology

The histological diagnosis of MTC was made on hematoxylin and eosin sections from formalin-fixed paraffin-embedded blocks by expert pathologists (Department of Surgical, Medical, Molecular Pathology and Clinical Area, University of Pisa), who were blinded to clinical data, according to WHO criteria. For each tumor, we applied the *American Joint Committee on Cancer (AJCC) Cancer Staging 8th edition*.

### 2.5. RET Somatic Mutation

In 254/529 (48.0%) patients, data about somatic genetic features were available thanks to previous next-generation sequencing (NGS) molecular profiling of tumoral tissue using our thyroid-specific custom panel that includes the whole coding region of *RET* and hot-spot portions of another 16 genes, as previously described [18].

### 2.6. Imaging Evaluation

In all patients, a neck ultrasound was performed at each clinical evaluation. Several instruments from Esaote Biomedica, Italy, were employed over time: the real time instrument Technos, with a 7.5–10 MHz linear transducer, from 2000 to February 2011; the doppler apparatus My Lab 50, with a 7.5–12-MHz linear transducer, from March 2011 to December 2018; the advanced doppler apparatus My Lab Twice, with a 7.5–12-MHz linear transducer, from January 2019 to study data lock. In case of lymph nodes suspicious for metastases at neck US, fine needle aspiration cytology associated with washing fluid Ct measurement was performed.

During the follow-up, according to good clinical practice, further imaging procedures [i.e., total body computed tomography scan with i.v. contrast medium, contrast-enhanced magnetic resonance imaging of the liver, and 18F-Fluorodeoxyglucose and/or Fluorine-18-L-dihydroxyphenylalanine Positron Emission Tomography/Computerized Tomography] were performed, if needed.

### 2.7. Response to Treatment Definition

At the first assessment after surgery, and at each clinical assessment during the follow-up, we divided the patients according to the clinical, biochemical, and imaging evaluations into excellent response (ER), biochemical incomplete response (BiR), and structural incomplete response (SiR). ER was defined in the case of undetectable values of Ct and negative imaging. BiR was defined in the case of detectable basal Ct and/or stimulated Ct values but without evidence of structural disease. SiR was defined in the case of tumoral lesions either detected by imaging or by positive biopsy [19].

### 2.8. Statistical Analysis

Data are presented as median values and interquartile range (IQR). The analysis was performed using SPSS Statistics for Windows (IBM Corp. Released 2017, IBM SPSS Statistics for Windows, Version 25.0. Armonk, NY, USA: IBM Corp). Mann–Whitney U test and Pearson’s chi-squared test (with Yates correction, if necessary) were performed. Disease-specific survival curves and the log-rank test were performed using the Kaplan–Meier analysis. A *p*-value of <0.05 was considered significant.

## 3. Results

### 3.1. Baseline Features

Epidemiological, biochemical, histological, and outcome data are reported in Table 1.

Most of the patients were female (288/529, 54.4%), and the median age at surgery was 55 years (IQR 45–66). The median BMI was 25.7 kg/m^2^, and 6/529 (1.1%), 223/529 (42.2%), 193/529 (36.5%), and 107/529 (20.2%) patients were classified as underweight, normal weight, overweight, and obese, respectively. According to our data, the prevalence of obesity among MTC patients was higher than the Italian general population, which is approximately 12%, as reported by official epidemiological data [20]. Since the number of patients in the underweight category was very low, we merged underweight and normal weight patients, therefore indicated as under/normal weight category for further analysis.

Before surgery, basal Ct values were available in 472/529 (89.2%) patients, and the median value was 136 pg/mL (IQR 31–730).

The median size of the primary tumor was 1.3 cm, and the tumor was > 4 cm in 38/529 (7.2%) patients. According to the eighth edition of *AJCC Cancer Staging*, most of the patients were classified as pT1a (40.1%), pT1b (28.4%), and pT2 (19.75%); conversely, pT3 (7.2%) and pT4 (4.5%) were less commonly diagnosed. Multifocality and minimal extrathyroidal extension (mETE) were observed in 76/529 (14.4%) and 89/529 (16.8%) patients, respectively.

Central compartment lymph node dissection was performed in 87.9% of patients (465/529). Lymph node metastases were observed in 216/529 (40.9%) patients, of whom 95/529 (18.0%) had lymph node metastases in the central compartment only (N1a), 23/529 (4.3%) in the latero-cervical compartment only (N1b), and 98/529 (18.5%) in both compartments. Distant metastases at diagnosis were present in 36/529 (6.8%) patients.

During a median follow-up time of 75 months (IQR 35–130), 12/529 (2.3%) patients were lost. Of the remaining, 92/517 (17.8%) were submitted for further treatment. Surgical reoperation for persistence/recurrence in the neck was performed in 35/517 (6.8%), local therapies (i.e., external beam radiation therapy) in 41/517 (7.9%), and systemic therapies in 55/529 (10.4%) patients. At the end of the follow-up, 119/517 (23.0%) patients experienced SiR; conversely, 307/517 (59.4%) patients had ER. In a median time of 98 (IQR 57–152) months, 39/517 (7.5%) patients passed away from causes related to MTC.

### 3.2. Epidemiological, Biochemical, and Pathological Data Across BMI Categories

Patients with obesity (61/107, 57%) and overweight (111/193, 57.5%) were more frequently older than 55 years compared to under/normal weight (91/229, 39.7%) (*p* < 0.01) (Table 2).

Moreover, male prevalence was lower in patients with obesity compared to overweight (45/107, 42.1%, vs. 107/193, 55.4%, *p* = 0.026); conversely, no sex differences were detected in the under/normal weight category (Table 2).

Preoperative Ct was available in 204/225 (89.5%), 170/193 (88.1%), and 97/107 (90.7%) patients with under/normal weight, overweight, and obesity, respectively. Patients with obesity had lower preoperative Ct levels compared to under/normal weight ones (median 69 vs. 167 pg/mL, *p* = 0.005); preoperative Ct levels did not differ between patients with obesity and overweight (median 69 vs. 109 pg/mL, *p* = 0.17) (Table 2).

In patients with obesity, tumor dimension (median 1.0, IQR 0.5–2.0 cm) was smaller than in overweight (median 1.3, IQR 0.7–2.5 cm, *p* = 0.018) and under/normal weight (median 1.5, IQR 0.8–2.5, *p* = 0.002). Also, a lower T stage was more common in patients with obesity compared to under/normal weight (Table 2, *p* = 0.002) but not in overweight patients (*p* = 0.128). Conversely, no differences in multifocality and mETE were detected across the different cohorts of patients (Table 2).

Regarding lymph node metastases, patients with obesity had an overall lower rate of N1 compared to under/normal weight (34/107, 31.8% vs. 101/229, 44.1%, *p* = 0.032) and to overweight patients (81/193, 41.9%, *p* = 0.082). Moreover, patients with obesity showed lower N stage compared to under/normal weight (*p* = 0.028) and overweight patients (*p* = 0.013). In contrast to lymph node involvement, the rate of distant metastasis did not differ across the three groups of patients (Table 2).

### 3.3. Genetic Data Across BMI Categories

Data about somatic mutations were available in 254/529 patients (48.0%). In this sub-group of patients, the median BMI was 25.3 (IQR 23.1–28.8) kg/m^2^, and 114/254 (44.9%), 91/254 (35.8%), and 49/254 (19.3%) patients were under/normal weight, overweight, and obese, respectively. Both median BMI (*p* = 0.649) and under/normal weight, overweight, and obese prevalences (*p* = 0.907) did not differ from the whole group (n = 529).

*RET* mutations were observed in 143/254 patients (56.3%). The prevalence of tumors with *RET* somatic mutations did not differ across BMI categories: 67/114 (58.8%) in under/normal weight, 50/91 (54.9%) in overweight, and 23/49 (53.1%) in patients with obesity (*p* = 0.37). Most of the *RET* positive cases (83/143—58.0%) carried the *RETM918T* somatic mutation, with no prevalence difference among the three groups. *RAS* mutations occurred in 13/254 (5.1%) without any different prevalence among patients with obesity (2/26, 7.6%), under/normal weight (5/47, 10.6%), and overweight (6/41, 14.6%, *p* = 0.66).

### 3.4. Follow-Up

**Figure 1:**
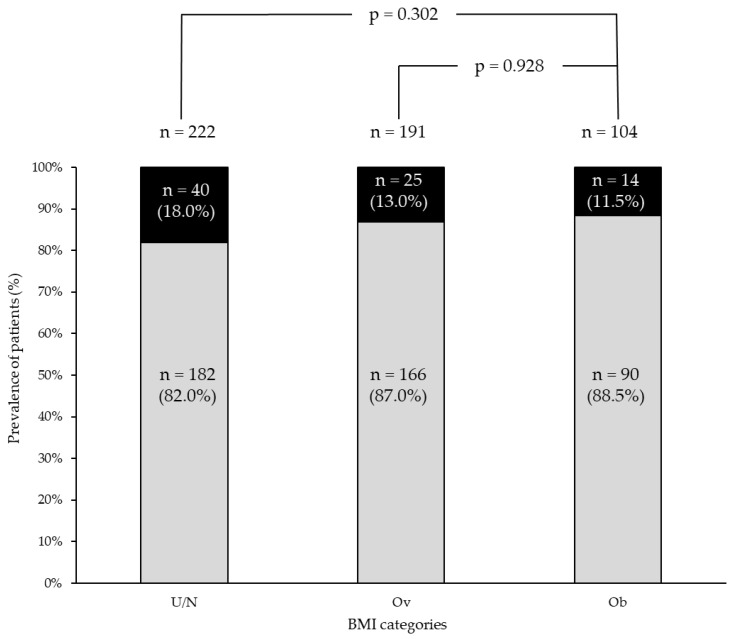
Prevalence of patients with (black bar) and without (light grey bar) a structural incomplete response at the first evaluation after surgery across the different BMI categories. U/N group: under/normal weight patients group; Ov group: overweight patients group; Ob group: obesity patients group.

At the first postoperative evaluation (median 5 months from surgery—IQR 3–7), 7/229 (3.0%), 2/193 (1.0%), and 3/107 (2.8%) patients in under/normal weight, overweight, and patients with obesity, respectively, were lost at the follow-up. In the remaining, clinical response did not differ significantly across the three groups (Figure 1).

Median follow-up time was 82 (IQR 39–136), 73 (IQR 37–130), and 66 (IQR 31–111) months in under/normal weight, overweight, and patients with obesity, respectively. No differences in the further therapies performed over time were detected among the three groups (16.7% in under/normal weight vs. 18.7% in overweight and 15.3% in patients with obesity, *p* = 0.73). However, when evaluating the type of further treatment performed (Table 3), re-operations in the neck were more frequently experienced by overweight patients (21/191, 11.0%) compared to under/normal weight (10/222, 4.5%, *p* = 0.015) and patients with obesity (4/104, 3.8%, *p* = 0.047).

**Figure 2:**
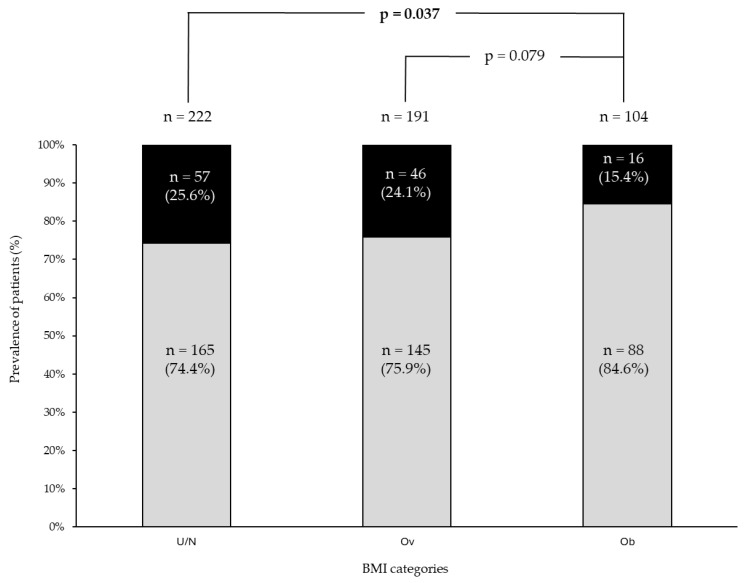
Prevalence of patients with (black bar) and without (light grey bar) a structural incomplete response at the end of follow-up across the different BMI categories. U/N group: under/normal weight patients group; Ov group: overweight patients group; Ob group: obesity patients group.

At the end of follow-up, SiR was significantly lower in patients with obesity (16/104, 15.4%) compared to under/normal weight (57/222, 25.6%, *p* = 0.037) and, although not statistically significant, it was also lower compared to overweight patients (46/191, 24.1%, *p* = 0.079) (Figure 2).

**Figure 3:**
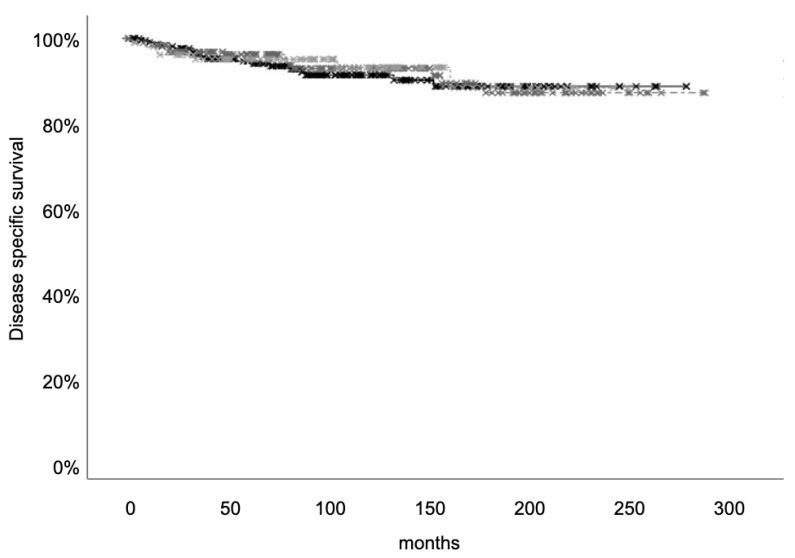
Disease-specific survival in under/normal (black line), overweight (dark grey line), and obese (light grey lines) patients.

Disease-specific survival was evaluated in a median time of 95.5 (IQR 57–153), 100 (IQR 57–153), and 101 (54–143) months in under/normal weight, overweight, and patients with obesity, respectively. No differences in the number of patients who died of MTC were found among the three categories [18/222 (8.1%) in under/normal weight, 14/191 (7.3%) in overweight, and 7/104 (6.75%) patients with obesity (*p* = ns)]. Moreover, disease-specific survival did not differ across the three groups (Figure 3).

## 4. Discussion

Obesity is a chronic relapsing disease marked by an excessive accumulation of body fat stemming from impaired energy balance mechanisms [21]. Excess adipose tissue induces various non-communicable diseases, such as diabetes, cardiovascular diseases, or cancer. In the US each year, 37,670 and 74,690 cancer cases were attributable to excess adipose tissue in men and women, respectively [22]. Globally, about 40% of cancer cases have been attributed to overweight and obesity [23], including meningioma, multiple myeloma, esophageal, gastric, colon–rectum, liver, gall bladder, pancreas, breast, corpus uteri, ovary, kidney, and thyroid cancer [1].

The role of obesity in DTC, particularly PTC, seems well-defined, with a clear impact of excess adiposity in increasing its prevalence [1] but with contrasting data about the impact on aggressiveness [5,8,9,10,11,12,13,14,15]. Conversely, the impact of excess adiposity on MTC prevalence and outcome is more ambiguous. This ambiguity may stem from the fact that MTC is significantly less common than other thyroid cancer histotypes, and it is challenging to collect enough cases to perform statistical analysis. Moreover, a distinction between hereditary and sporadic cases should be made to evaluate the association with obesity. The aim of the present study was to solve this uncertainty by evaluating a large series of consecutive patients with sporadic MTC and correlating BMI value at diagnosis with the clinical presentation and outcome of the disease.

In this series of sporadic MTC patients, the prevalence of subjects with obesity was higher than that in the Italian general population but not different from that in DTC [15], hypothesizing a potential role of excess adipose tissue in MTC development. Conversely, Kitahara et al. performed a meta-analysis of 22 prospective studies evaluating patients with all kinds of thyroid cancers, also including 100 MTC cases. In this meta-analysis, patients with obesity did not experience an increased risk of MTC [5]. In contrast, Engeland et al. observed that the risk of MTC even decreased with increasing BMI [6]. However, in both aforementioned studies, the number of patients with MTC was rather lower, without any distinction between sporadic and hereditary cases. In this regard, we think that the high number of patients included in our study and the enrollment of only sporadic MTCs could shed new light on a potential role of obesity on MTC.

Excess adipose tissue has been associated with a worse prognosis in many cancers [24]. In this study, we evaluated, for the first time, a possible association between BMI and sporadic MTC aggressiveness. MTC patients with obesity presented a less extensive disease characterized by lower preoperative Ct levels, smaller tumors, and lower T and N stages compared to over- and under/normal-weight patients. In line with that, patients with obesity presented less frequent structural disease at the end of follow-up compared to over- and under/normal-weight ones. In contrast, in DTC, a conflicting association was reported between BMI and tumor aggressiveness; some authors observed an increased aggressiveness in patients with obesity [5,12], which was not confirmed by others [11,15]. Moving to other cancers, such as clear-cell renal cell carcinoma, patients with obesity showed less frequent advanced-stage disease compared with normal-weight ones [25]. Likewise, a less aggressive disease has been reported in patients with obesity suffering from colorectal cancer [26]. In this regard, the most frequent medical evaluations experienced by patients with obesity, potentially leading to an early detection of MTC, cannot be excluded. At the same time, excess adipose tissue may have a direct impact on MTC, modulating tumor infiltration or cancer metabolism, as observed in other tumors [27].

Patients with obesity are usually associated with lower disease-specific survival [2]. In this regard, Petrelli et al. analyzed data coming from 203 studies with 6,320,365 participants and observed a reduction in overall and cancer-specific survival in patients with obesity [3]. On the other hand, in the case of lung cancer, renal cell carcinoma, or melanoma, patients with obesity had better survival outcomes compared with those without obesity [3]. In DTC, data regarding survival are conflicting. Gąsior-Perczak et al. found no association between BMI and overall survival [13], while Kitahara et al. found an association between baseline BMI and increased risk of thyroid cancer mortality [5]. In sporadic MTC, we did not observe any differences in survival in patients with obesity compared to those with overweight or under/normal weight. In line with that, we did not observe any differences in distant metastasis across BMI categories. So far, we do not have any other study on this specific issue to be compared with ours, and it would be interesting to verify these data in other larger series.

Recent studies showed that patients with obesity have a specific genetic signature. In breast cancer, Nguyen et al. showed genomic alterations differentially prevalent in patients with obesity compared to normal-weight ones [28]. In lung adenocarcinoma, patients with obesity showed a lower prevalence of *EGFR* and a higher prevalence of *KRAS* mutations [29]. Likewise, in PTC, patients with obesity harbored a higher prevalence of *BRAF* mutations compared to over- and normal-weight ones [30]. In our series, we did not observe any different prevalences of somatic *RET* and *RAS* mutations across BMI categories. Indeed, new evidence showed a crucial role of the *RET* gene in weight control. In the hindbrain, the binding of complex RET-GFRAL with its ligand GDF15 causes weight loss via modulating food intake [31]. However, it is conceivable that in sporadic MTC, in which *RET* mutations are limited to tumoral cells, this mechanism is not impaired. It would be of interest to verify *RET* mutations’ impact on weight control in subjects with hereditary MTC, where the *RET* mutation is ubiquitously expressed.

Our study has some limitations. The evaluation of excess adiposity was based on BMI, although it is not able to fully recapitulate body composition and fat distribution. Alternative anthropometric measurements have been proposed to provide a more comprehensive assessment of obesity, such as neck circumference and waist-to-hip ratio, but they are more complex to measure [32]. On the other hand, BMI was calculated in each patient by physicians after weight and height measurement, and this should be considered a strength since, in other series, BMI was reported by the patient and was not always reliable [33]. Another limitation could be that we did not evaluate the dynamic change in BMI during the follow-up. However, the aim of the current research was to evaluate the prevalence of obesity in MTC patients and the potential influence of BMI at diagnosis on MTC aggressiveness and outcome. Finally, the retrospective nature of the research could be considered a limitation, but we think that the high number of enrolled patients, the long follow-up, and the prospective collection of data could overcome this limitation.

## 5. Conclusions

Our study identified a higher prevalence of patients with obesity among a large series of sporadic MTC compared to that reported in the general population. However, the aggressiveness of the disease was lower in patients with obesity at diagnosis, resulting in a lower need for further surgical therapies during the follow-up, a lower prevalence of structural disease at the end of the follow-up, and survival similar to that of normal weight MTC. No difference in *RET* somatic mutations was observed across different BMI categories.

## Figures and Tables

**Table 1:** Epidemiological, biochemical, histological, and outcome of the whole cohort of patients.

Epidemiologic Features		Total Cohort *n* = 529 (%)
Gender	M	241 (45.6)
	F	288 (54.4)
Age at diagnosis (years)	Median, IQR	55 (45–66)
	≤55 years	266 (50.3)
	>55 years	263 (49.7)
Biochemical features		
Preoperative calcitonin (*n* = 427)	Median, IQR	136 (31–730)
Histology		
Tumor size (cm)	Median, IQR	1.3 (0.7–2.4)
	≤1	208 (39.5)
	1.1–4	283 (53.5)
	>4	38 (7.2)
T stage according to AJCC 8th edition	T1a	212 (40.1)
	T1b	148 (28.4)
	T2	104 (19.7)
	T3	38 (7.2)
	T4	24 (4.5)
	Tx	1 (0.2)
Tumor Multifocality	Yes	76 (14.4)
	No	453 (85.6)
Tumor mETE	Yes	89 (16.8)
	No	440 (83.2)
Lymph node metastasis	Yes	216 (40.9)
	No	313 (59.1)
Central compartment dissection	Yes	465 (87.9)
	No	64 (12.1)
N stage according to AJCC 8th edition	Nx	47 (8.9)
	N0	266 (50.2)
	N1a	95 (18.0)
	N1b	121 (22.9)
M stage according to AJCC 8th edition	Mx/M0	493 (93.2)
	M1	36 (6.8)
Staging according to AJCC 8th edition	Stage I	252 (47.6)
	Stage II	57 (10.8)
	Stage III	91 (17.2)
	Stage IV	129 (34.4)
Follow-up data		
Disease status at end of follow-up	ER	313 (59.1)
	BiR	85 (16.1)
	SiR	119 (22.5)
	Unknown	12 (2.3)
Follow-up (time, months)	Median, IQR	75 (35–130)
Further treatments	Yes	92 (17.5)
	No	437 (82.5)
Death for disease	Yes	39 (7.4)
	No	490 (92.6)
Disease survival specific Time (time, months)	Median, IQR	98 (57–152)

IQR: interquartile range; mETE: minimal extrathyroidal extension; AJCC: American Joint Committee on Cancer.

**Table 2:** Epidemiological, biochemical, histological, and outcome across BMI categories.

		U/N Group n = 229 (%)	Ov Group n = 193 (%)	Ob Group n = 107 (%)	*p*-Value U/N vs. Ov Group	*p*-Value U/N vs. Ob Group	*p*-Value Ov vs. Ob Group
**Epidemiologic features**							
**Sex**	Male	89 (38.9)	107 (55.4)	45 (42.1)	**0.001**	0.57	**0.026**
	Female	140 (61.1)	86 (44.6)	62 (57.9)			
**Age at diagnosis (years)**	Median, IQR	52 (43–63)	58 (47–65)	58 (48–69)	**0.002**	**0.001**	0.320
	≤55 years	138 (60.3)	82 (42.5)	46 (43.0)	**<0.001**	**<0.001**	0.933
	>55 years	91 (39.7)	111 (57.5)	61 (57.0)			
**Biochemical features**							
**Preoperative calcitonin (n = 427)**	Median, IQR	167 (44–822)	109 (30–766)	69 (20–493)	0.09	**0.005**	0.17
**Histology**							
**Size (cm)**	Median, IQR	1.5 (0.8–2.5)	1.3 (0.7–2.5)	1.0 (0.5–2.0)	0.48	**0.002**	**0.018**
	≤1	80 (35.1)	73 (38.0)	55 (51.4)	0.768	**0.019**	**0.003**
	1.1–4	127 (55.7)	104 (54.2)	50 (46.7)			
	>4	21 (9.2)	15 (7.8)	2 (1.9)			
**T stage according to AJCC 8th edition**	T1a	78 (34.1)	78 (40.4)	56 (52.3)	0.19	**0.002**	0.128
	T1b	72 (31.4)	52 (26.9)	26 (24.3)			
	T2	41 (17.9)	42 (21.7)	19 (17.8)			
	T3	25 (11.0)	11 (5.7)	2 (1.8)			
	T4	12 (5.2)	10 (5.2)	2 (1.8)			
	Tx	1 (0.4)	0	0			
**Multifocality**	Yes	33 (14.4)	25 (13.0)	18 (16.8)	0.665	0.566	0.360
	No	196 (85.6)	168 (87.0)	89 (83.2)			
**mETE**	Yes	42 (18.3)	35 (18.1)	12 (11.2)	0.956	0.098	0.114
	No	187 (81.7)	158 (81.9)	95 (88.8)			
**Lymph node metastasis**	Yes	101 (44.1)	81 (41.9)	34 (31.8)	0.659	**0.032**	0.082
	No	128 (55.9)	112 (58.1)	73 (68.2)			
**Central compartment dissection**	Yes	206 (90.0)	170 (88.1)	89 (83.2)	0.538	0.077	0.236
	No	23 (10.0)	23 (11.9)	18 (16.8)			
**N stage according to AJCC 8th edition**	Nx	17 (7.4)	13 (6.7)	17 (15.9)	0.641	**0.028**	**0.013**
	N0	111 (48.5)	99 (51.4)	56 (52.3)			
	N1a	46 (20.1)	30 (15.5)	19 (17.8)			
	N1b	55 (24.0)	51 (26.4)	15 (14.0)			
**M stage according to AJCC 8th edition**	Mx/M0	213 (93.0)	180 (93.3)	100 (93.5)	0.919	0.935	0.859
	M1	16 (7.0)	13 (6.7)	7 (6.5)			
**Staging according to AJCC 8th edition**	Stage I	102 (44.4)	89 (46.2)	61 (57.1)	0.776	0.177	0.166
	Stage II	26 (11.4)	22 (11.4)	9 (8.4)			
	Stage III	43 (18.8)	29 (15.0)	18 (16.8)			
	Stage IV	58 (25.4)	53 (27.4)	19 (17.7)			

U/N group: underweight/normal weight patients group; Ov group: overweight patients group; Ob group: obesity patients group; IQR: interquartile range; mETE: minimal extrathyroidal extension; AJCC: American Joint Committee on Cancer. Bold characters in the *p*-value columns represents statistically significant results.

**Table 3:** Prevalence of further therapies performed after initial surgical treatment.

		U/N Group n = 229 (%)	Ov Group n = 193 (%)	Ob Group n = 107 (%)	*p*-Value U/N vs. Ov Group	*p*-Value U/N vs. Ob Group	*p*-Value Ov vs. Ob Group
**Further therapies**							
**Cervical surgical therapies**	Yes	10 (4.5)	21 (11.0)	4 (3.8)	**0.015**	1.000	**0.047**
	No	212 (95.5)	170 (89.0)	100 (96.2)			
**Local therapies against distant metastases**	Yes	15 (6.8)	19 (9.9)	7 (6.7)	0.283	1.000	0.398
	No	207 (93.2)	172 (90.1)	97 (93.3)			
**Systemic therapies**	Yes	29 (13.1)	18 (9.4)	8 (7.7)	0.246	0.191	0.673
	No	193 (86.9)	173 (90.6)	96 (92.3)			

U/N group: under/normal weight patients group; Ov group: overweight patients group; Ob group: obesity patients group. Bold characters in the *p*-value columns represents statistically significant results.

## Data Availability

The raw data supporting the conclusions of this article will be made available by the authors on request.

## Abbreviations

The following abbreviations are used in this manuscript:

AJCC	American Joint Committee on Cancer
ATC	Anaplastic thyroid cancer
BiR	Biochemical incomplete response
BMI	Body mass index
Ct	Calcitonin
DTC	Differentiated thyroid cancer
ER	Excellent response
FTC	Follicular thyroid cancer
IQR	Interquartile range
mETE	Minimal extrathyroidal extension
MTC	Medullary thyroid cancer
NGS	Next-generation sequencing
Ob	Obesity group
Ov	Overweight group
PDTC	Poorly differentiated thyroid cancer
PTC	Papillary thyroid cancer
RET gene	Rearranged during transfection gene
SiR	Structural incomplete response
U/N	Under/normal weight group
